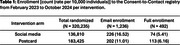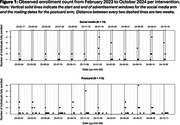# An interrupted time‐series design experiment of research registry recruitment interventions

**DOI:** 10.1002/alz70859_103378

**Published:** 2025-12-25

**Authors:** Thuy V Lu, Megan G Witbracht, Maricela Cruz, Dan Hoang, Amy J.H. Kind, Josh D Grill, Daniel L Gillen

**Affiliations:** ^1^ The UC Irvine Institute for Memory Impairments and Neurological Disorders, Irvine, CA USA; ^2^ University of California, Irvine, Irvine, CA USA; ^3^ Institute for Memory Impairments and Neurological Disorders, University of California, Irvine, Irvine, CA USA; ^4^ Kaiser Permanente Washington Health Research Institute, Seattle, WA USA; ^5^ University of Washington, Seattle, WA USA; ^6^ Center for Health Disparities Research, University of Wisconsin School of Medicine and Public Health, Madison, WI USA; ^7^ University of Wisconsin, Madison, WI USA

## Abstract

**Background:**

We conducted an interrupted time series design experiment to assess the effectiveness of interventions to recruit individuals from disadvantaged neighborhoods in Orange County, California to the Consent‐to‐Contact (C2C) registry at UC Irvine (UCI). This study quantified the effect of social media and traditionally mailed postcard advertisement interventions on C2C enrollment rates.

**Method:**

From February 2023 to October 2024, we launched advertisements on Facebook/Instagram and sent postcard to randomly sampled census block groups in 12 periods with 4‐week washout periods between each. We quantified individuals enrolled in the C2C at two levels: (1) email enrollment to receive information on research studies at UCI and (2) full enrollment to enable matching and referral to research studies. Using interrupted time series methods, we estimated the impact of each intervention on enrollment. We a priori defined three two‐week periods: before, during, and after intervention. The intervention period began on the earlier start date of either the social media or postcard interventions. We estimated the change point when the intervention initiated an effect and compared the post‐change point mean function to the projected pre‐change point mean function to assess changes in the enrollment rate.

**Result:**

Table 1 presents the raw enrollment count (rate) per intervention. Figure 1 shows the observed enrollment count across time per intervention. Enrollment rates per 10,000 individuals differed between pre‐ and post‐intervention periods: 0 versus 5.41 in the social media arm, and 0.22 versus 6.16 in the postcard arm. The social media arm yielded 72 confirmed enrollments during the first intervention week, with none before or after. For postcard intervention, we estimated the effect on enrollment initiated on average 4 days after the start of pre‐specified intervention periods. We estimated that enrollment rate was 19‐fold higher at the estimated change point comparing the post‐change point mean function to the projected pre‐change point mean function (95% CI: 6.0403, 62.3953).

**Conclusion:**

We observed immediate enrollments after the social media advertisement implementation. Mailed postcard intervention was estimated to impact C2C enrollment soon after the postcards were mailed. Our findings provide insights into how the interventions may perform in recruiting individuals to research registries.